# Genome-Wide Identification and Expression Analysis of the Aquaporin Gene Family in the Qinghai Toad-Headed Agama (*Phrynocephalus vlangalii*) and Responses to Acute Cold Stress

**DOI:** 10.3390/biology14121755

**Published:** 2025-12-07

**Authors:** Yurong Zhang, Ping Yang, Xinyang Li, Jia Wang

**Affiliations:** Xinjiang Key Laboratory for Ecological Adaptation and Evolution of Extreme Environment Organisms, College of Life Sciences, Xinjiang Agricultural University, Urumqi 830052, China; zzhangyr07@163.com (Y.Z.); zkayp1314@163.com (P.Y.); lxy010718@163.com (X.L.)

**Keywords:** aquaporins, *Phrynocephalus vlangalii*, temperature, expression analysis, adaptation

## Abstract

The aquaporin (AQP) gene family plays a critical role in water balance and osmotic regulation, yet its function and regulatory mechanisms in plateau reptiles remain poorly understood. In this study, we systematically identified 10 AQP genes in the Qinghai toad-headed agama (*Phrynocephalus vlangalii*) based on whole-genome data, and conducted a comprehensive analysis of their physicochemical properties, phylogenetic relationships, conserved domains, gene structures, and expression patterns. The results showed that the AQP genes of *P. vlangalii* are predicted to localize to the plasma membrane and exhibit significant tissue-specific expression, with the highest levels detected in the kidney and liver. Under low-temperature stress, multiple AQP genes displayed dynamic expression patterns during the stress and recovery phases. Specifically, *AQP0*, *AQP2*, and *AQP5* were persistently downregulated in the liver, kidney, and brain, whereas *AQP3*, *AQP7*, and *AQP9* were initially upregulated during early cold exposure but significantly downregulated during recovery, suggesting their coordinated roles in energy metabolism and water conservation. This study provides evidence supporting the involvement of the AQP gene family in the adaptation of *P. vlangalii* to the cold and arid plateau environment, providing new insights into the regulatory mechanisms of water metabolism in reptiles.

## Simple Summary

*Phrynocephalus vlangalii* is a unique oviparous lizard species in China. Previous field observations have revealed that *P. vlangalii* can enter a state of cold torpor with apparent freezing tolerance during non-hibernation seasons when triggered by sudden temperature drops and recover normal physiological activity upon warming. Tolerance to acute cold stress is exceptionally rare among reptiles, and the underlying molecular physiological mechanisms remain unclear. Aquaporins (AQPs) are a family of transmembrane proteins that play essential roles in maintaining water balance, regulating cellular functions, and supporting energy metabolism. Studies on freeze-tolerant frogs and insects have suggested that AQPs may play a significant role in whole-body water redistribution during low-temperature stress. We hypothesize that the remarkable tolerance to acute cold stress in *P. vlangalii* critically depends on the coordinated action of its AQP family members at the molecular level. Nevertheless, the regulation of water transport in *P. vlangalii* remains poorly understood, and the roles of different AQPs under varying temperature conditions are still unclear. This study aims to characterize the AQP gene family in *P. vlangalii* and analyze its tissue-specific expression patterns. By investigating how AQPs might regulate internal water balance, this research seeks to identify the molecular mechanisms of water metabolism underpinning extreme climate adaptation, providing insights for conserving this and other plateau species.

## 1. Introduction

Aquaporins (AQPs) are a family of transmembrane proteins ubiquitously present in all life forms, from bacteria to humans [1]. They form highly selective channels in cell membranes that primarily mediate the efficient transmembrane transport of water molecules, while also permitting the passage of certain small solutes (e.g., glycerol, urea) [2,3]. These proteins play essential roles in maintaining water balance, regulating cellular functions, and supporting energy metabolism [4,5,6]. AQPs are composed of approximately 300 amino acid residues and typically contain six transmembrane helices. They assemble into three distinct structural forms: monomer, dimer, and tetramer. Through these structures, pores are formed in the plasma membrane. These pores consist of five connecting loops, together with the six transmembrane helices and the N- and C-termini regions, collectively facilitating controlled transport [7].

AQPs have been extensively characterized in various species. For example, *AQP0* has been found to be expressed in the lenses of mammals such as rabbits and cattle, as well as in fish and birds. *AQP1*, *AQP2*, *AQP3*, and *AQP4* were highly expressed in the kidneys of mammals. *AQP5* was predominantly localized to salivary glands, with lower levels detected in sweat and lacrimal glands. *AQP6* was mainly expressed in the central nervous system and kidneys, while *AQP8* showed elevated expression in the liver [8,9,10,11]. Currently, studies on the role of AQPs in reptiles remain limited. Evidence suggests that most reptiles possess 10 AQP genes, which may reflect functional complementarity unique to this group [12]. In lizards, *AQP2*, *AQP5*, and *AQP6* are implicated in important roles in the skin, bladder, salt glands, and kidneys [13]. In particular, *AQP2* and *AQP6* are predicted to be crucial for adaptation to terrestrial environments, with identified positive selection sites potentially associated with water conservation [14]. The expression of *AQP4* in the central nervous system also contributes to water and ion homeostasis in reptiles [15]. A study on *Eremias multiocellata* revealed seasonal dynamics in AQP expression: *AQP1* levels were higher in the small intestine but lower in the large intestine during winter, whereas *AQP2* remained highly expressed despite reduced renal function. This asynchronous regulation of AQP genes highlights their complementary relationship with the organismal physiological needs [16].

The Qinghai–Tibet Plateau (QTP), the largest and highest in the world, has an average elevation of approximately 4000 m and is often referred to as the “Roof of the World.” Its unique geography, complex topography, extreme climate, and rich biodiversity support a wide variety of species, including fish, birds, mammals, reptiles, amphibians, and plants [17]. The Qinghai toad-headed agama, *Phrynocephalus vlangalii* (Squamata: Lacertilia: Agamidae), is a unique oviparous lizard species in China and the most widely distributed lizard on the QTP. Its range spans the entire Qaidam Basin and surrounding areas, covering elevations from 2000 m to 4500 m, making it an ideal model for studying plateau adaptation [18].

Current research on the physiological adaptations of *P. vlangalii* has primarily focused on heat tolerance under hypoxic conditions. Molecular studies have employed gene sequencing to identify dominant gut microbiota at different altitudes [19], and multi-platform metabolomics has been applied to characterize metabolic profiles and identify differential metabolites and pathways related to high-altitude adaptation [20]. Previous field observations have revealed that *P. vlangalii* can enter a state of apparent freezing during non-hibernation seasons when triggered by sudden temperature drops and recover normal physiological activity upon warming. The capacity to withstand acute cold stress is exceptionally rare among reptiles, and the underlying molecular physiological mechanisms remain unclear, making it a highly attractive research topic in the life sciences. Studies on freeze-tolerant frogs and insects have suggested that AQPs may play a significant role in whole-body water redistribution during low-temperature stress, and their expression levels are closely associated with insect cold hardiness and dehydration tolerance [4,21,22,23]. We hypothesize that the remarkable tolerance to acute cold stress in *P. vlangalii* critically depends on the coordinated action of its AQP family members at the molecular level: (i) classical aquaporins (e.g., *AQP0*, *AQP2*, *AQP5*) are generally downregulated to reduce transmembrane water flux, preventing ice crystal damage and cellular dehydration, and (ii) aquaglyceroporins (e.g., *AQP3*, *AQP7*, *AQP9*) are specifically upregulated in metabolically active tissues (e.g., liver) to facilitate glycerol transport, supporting cryoprotection and energy metabolism. Nevertheless, the regulation of water transport in *P. vlangalii* remains poorly understood, and the roles of different AQPs under varying temperature conditions are still unclear. This study aims to characterize the AQP gene family in the *P. vlangalii* and analyze the expression patterns of its members across different tissues. By investigating how the *P. vlangalii* regulates internal water balance to adapt to complex and variable environments, this research seeks to uncover the molecular mechanisms of water metabolism regulation. These findings provide insights into physiological adaptation under extreme climate conditions and support conservation strategies for this and other plateau species.

## 2. Materials and Methods

### 2.1. Sources of Experimental Animals and Tissue Sample Collection

A total of 12 healthy adult *P. vlangalii* were collected in August 2024 from Qiemo, Xinjiang, China (37°24′07″ N; 85°42′11″ E; 2836 m). The sex of all participants was recorded, and it was confirmed that they were all adults. Prior to the experiment, all individuals were acclimated under standard animal housing conditions, and their health was monitored daily to ensure only healthy subjects participated. To investigate the effects of acute low-temperature stress on this species, the individuals were randomly divided into three groups (n = 4 per group). One group was maintained in the animal room without any treatment and designated as the control group. Another group was subjected to cooling at a rate of 3 °C/h until reaching −2 °C, maintained at this temperature for 6 h, and then immediately euthanized; this group was designated as the cold stress group. The third group underwent the same cooling procedure followed by rewarming to room temperature at 3 °C/h and was then euthanized, serving as the cold stress recovery group. Tissue samples from the heart, liver, brain, kidney, lung, and muscle were collected, labeled, placed into RNA-free microtubes, and stored at −80 °C for subsequent analysis.

### 2.2. Identification and Characterization of the AQP Gene Family

To identify AQP genes in *P. vlangalii*, we first retrieved protein sequences of 10 AQPs from five model species from the NCBI database. These sequences were aligned using BLAST (https://blast.ncbi.nlm.nih.gov/Blast.cgi), and the top-scoring hits were selected for subsequent analysis. We obtained the genome assembly of *P. vlangalii* from the CNCB (gwh assembly: Phrvla1.0), including protein and DNA sequences as well as GFF annotation files, from which corresponding protein sequences were extracted. A total of AQP genes were identified.

Physicochemical properties of the encoded proteins, such as amino acid composition, molecular weight, isoelectric point, instability index, aliphatic index, and grand average hydropathy (GRAVY), were predicted using the ProtParam tool (https://web.expasy.org/protparam/). Subcellular localizations of the AQP proteins were inferred with WOLF-PSORT (https://wolfpsort.hgc.jp/).

For evolutionary analysis, AQP RNA sequences were aligned using MEGA11 (version 11.0.13), and a phylogenetic tree was constructed by the Neighbor-Joining (NJ) method with 1000 bootstrap replicates [24]. Protein domains were analyzed via the CD-Search tool (https://www.ncbi.nlm.nih.gov/Structure/cdd/wrpsb.cgi?tdsourcetag) on the NCBI. Conserved motifs were identified by submitting the AQP protein sequences to the MEME website (https://meme-suite.org/meme/). The analysis was performed using the following parameters: maximum number of motifs = 10, and sites were set to Zero or One Occurrence Per Sequence (zoops). Results were visualized using TBtools (version 2.143) [25].

### 2.3. Phylogenetic Analysis of the AQP Family

A phylogenetic tree was constructed using protein sequences from *P. vlangalii* and four reference species, *Homo sapiens*, *Caenorhabditis elegans*, *Mus musculus*, and *Xenopus laevis*, obtained from the UniProt database. Sequences from all five species were combined and aligned using MUSCLE in MEGA11 (version 11.0.13). The resulting alignment was trimmed to remove poorly aligned positions. The best-fit evolutionary model was determined using the LG model with gamma-distributed rate heterogeneity (LG + G). Under the LG + G model, a maximum likelihood tree was constructed using MEGA, with branch support evaluated via 1000 bootstrap replicates. Final visualization and annotation of the tree were completed using iTOL (https://itol.embl.de/).

### 2.4. Structural Prediction of the AQP Proteins

Secondary structures were predicted using the SOPMA online server (https://npsa-prabi.ibcp.fr/cgi-bin/npsa_automat.pl?page=npsa%20_sopma.html) with the following parameters: window width = 17, similarity threshold = 8, and number of states = 4, with results categorized into α-helix, β-turn, extended strand, and random coil. Tertiary structures were modeled via the SWISS-MODEL server (https://swissmodel.expasy.org/), selecting templates based on high sequence identity, coverage, and a GMQE score > 0.7. Model quality was assessed using GMQE (range 0–1) and QMEAN scores. The tertiary structure characteristics of all identified AQP proteins in *P. vlangalii* are summarized in Table S1. All quality metrics and template information are provided in Supplementary Table S2. Transmembrane topology was predicted using TMHMM 2.0 (https://services.healthtech.dtu.dk/services/TMHMM-2.0/).

### 2.5. RNA Extraction and cDNA Synthesis

Tissue samples (0.2–0.3 g) were homogenized in liquid nitrogen using a tissue grinder. Total RNA was extracted with the RNA pure Tissue & Cell Kit (CW0560S, Cwbio, Taizhou, China). RNA integrity was verified by agarose gel electrophoresis. Qualified RNA was divided into two applications: one portion was reverse-transcribed into cDNA, and the other was reserved for sequencing.

### 2.6. Primer Design and Quantitative PCR (qPCR)

A total of 12 pairs of gene-specific primers (Table 1) were designed from cDNA sequences using Primer Premier (version 5.0.0) and validated for qPCR. In these experiments, ATCB was used as a reference gene to normalize expression values. qPCR was performed in a 10 μL reaction mixture containing 0.5 μL template DNA, 5 μL 2 × M5 HiPer SYBR Premix EsTaq (with Tli RNaseH), 3.5 μL ddH_2_O, and 0.5 μL of each forward and reverse primer. The thermal cycling conditions were: 95 °C, 5 min; 40 cycles of 95 °C, 30 s; 60 °C, 30 s; 72 °C, 30 s [26]. Besides, The original quantitative PCR (qPCR) data for all samples are compiled in Table S3. Furthermore, the melting curve analyses for both reference and target AQP genes, confirming amplification specificity, are presented in Figure S1. The complete original dataset used for the Analysis of Variance (ANOVA) is available in Table S4.

### 2.7. Statistical Analysis

Cycle threshold (CT) values were used to calculate ΔCT and ΔΔCT. Relative gene expression was determined using the 2^−ΔΔCT^ method [27]. Statistical analyses were conducted with Graphpad Prism (version 9.1.0) software using Two-way ANOVA where appropriate. The Benjamini–Hochberg FDR correction was applied to all post hoc comparisons derived from the Linear Mixed-Effects model (see below). In the Results section and figure legends, we report FDR-adjusted *p*-values.

## 3. Results

### 3.1. Identification and Physicochemical Properties of the AQP Gene Family

A total of 10 AQP genes were identified in *P. vlangalii* and named Pv*AQP0*-Pv*AQP9*. Analysis of their physicochemical properties revealed that the AQP proteins vary in length from 243 (*AQP8*) to 312 (*AQP4*) amino acids (Table 2), with molecular weights ranging from 26,088.66 Da (*AQP8*) to 33,801.56 Da (*AQP4*). The isoelectric points (pI) of these proteins spanned from 5.61 (*AQP8*) to 7.77 (*AQP6*), reflecting a broad range of charge characteristics under physiological conditions. The instability index ranged from 24.51 to 47.92, with only *AQP2* exhibiting a value greater than 40, indicating that the majority of these AQP proteins are structurally stable. Subcellular localization predictions confirmed that all AQP members are localized to the plasma membrane.

### 3.2. Gene Structure Analysis and Conserved Motif Composition of AQP Gene Family

**Figure 1:**
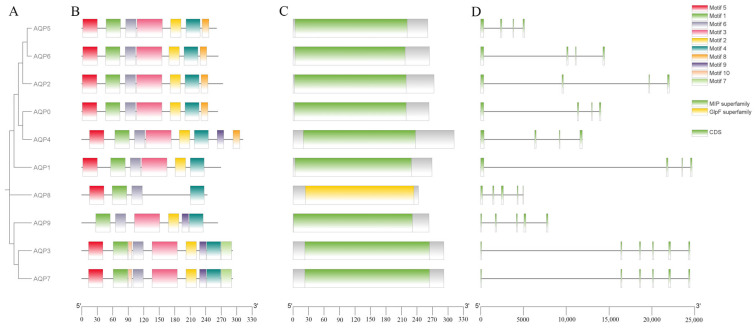
Characterization of AQPs in *Phrynocephalus vlangalii*. (**A**) Phylogenetic tree, displaying the evolutionary relationships among AQP genes in *P. vlangalii*. (**B**) Conserved motif composition of AQP proteins. Each motif is represented by a different colored box. (**C**) Domain architecture of AQP genes. (**D**) Exon–intron organization of AQP genes, where green boxes indicate coding sequences (CDS) and black lines represent introns.

A phylogenetic tree was constructed using the AQP amino acid sequences of *P. vlangalii* (Figure 1A). The tree resolved into three distinct clades: one comprising *AQP0*, *AQP1*, *AQP2*, *AQP4*, *AQP5*, *AQP6*; a second consisting of *AQP3*, *AQP7* and *AQP9*; and a third containing *AQP8* alone. Phylogenetically, most AQPs belong to the MIP superfamily, with *AQP8* classified into the GlpF superfamily, corresponding to the classical aquaporin subfamily and the water–glycerol channel subfamilies, respectively.

The results of the conserved motif analysis are shown in Figure 1B. A total of 10 consensus motifs were identified in the AQP gene family. All AQP members contain Motifs 1, 4, and 6. Motifs 2, 3, and 5 are present in 9 members, while the remaining motifs show a more limited distribution. Notably, Motifs 1, 2, 3, 5, and 9 exhibit lower E-values, indicating higher conservation significance. The first six motifs represent the largest number of motifs across all AQP proteins.

The gene structure analysis, as illustrated in Figure 1C, revealed distinct organizational patterns among AQP genes. Six genes (*AQP1*, *AQP2*, *AQP3*, *AQP7*, *AQP8* and *AQP9*) display complex structures with multiple introns interrupting their coding sequences. In contrast, *AQP5*, *AQP4* and *AQP6* possess relatively simple gene architectures with fewer introns. One gene (*AQP0*) was excluded from this structural analysis due to incomplete genome annotation.

Together, these findings demonstrate that while the AQP gene family in *P. vlangalii* maintains conserved motif characteristics, it exhibits considerable diversity in gene structure (Figure 1D). As shown, the CDSs of *AQP1*, *AQP2*, *AQP3*, *AQP7*, *AQP8*, and *AQP9* are dispersed across the gene locus and interrupted by multiple introns, reflecting a relatively complex gene structure that requires extensive post-transcriptional splicing. In contrast, the CDS segments of the *AQP5* gene are more concentrated, indicative of a simpler gene structure. This compact organization may support a more stable expression pattern during translation, potentially facilitating rapid and consistent functionality as a water channel protein in specific tissues such as alveolar epithelium, thereby contributing to fluid homeostasis. The CDS distributions of *AQP4* and *AQP6* display an intermediate spacing pattern, suggesting a moderate level of structural complexity. Such an arrangement could allow these proteins to balance structural flexibility with functional stability during water and small solute transport, enabling adaptation to varying physiological conditions while maintaining structural integrity. These structural features are consistent with their regulatory roles in tissues, including the central nervous system and kidneys.

### 3.3. Phylogenetic Analysis of AQP Gene Family

**Figure 2:**
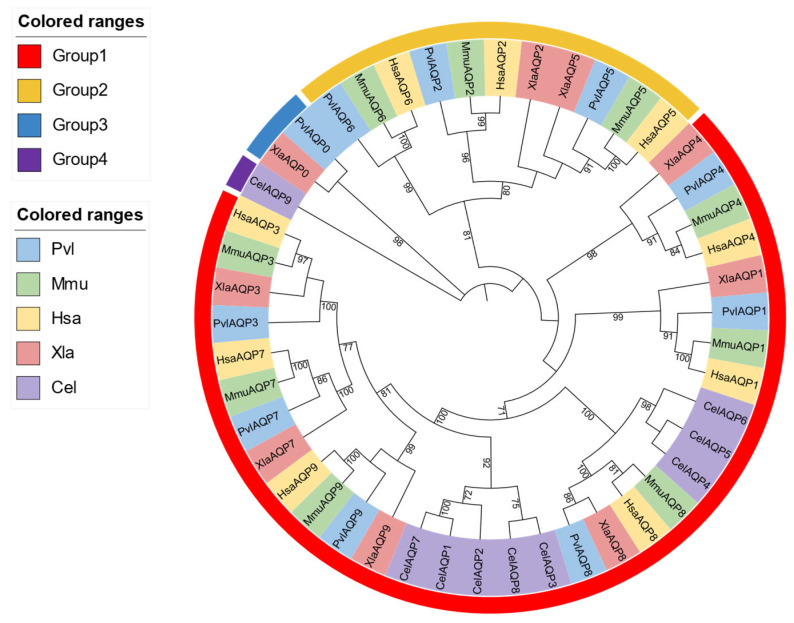
Phylogenetic tree of the AQP gene family. Note: Groups 1–4 are color-coded to indicate the evolutionary groupings of the AQP proteins. The tree includes AQP protein sequences from *P. vlangalii* (blue), *Mus musculus* (green), *Homo sapiens* (yellow), *Xenopus laevis* (pink), and *Caenorhabditis elegans* (purple).

The phylogenetic analysis revealed distinct evolutionary relationships among the AQP genes of *P. vlangalii* with representative model organisms (Figure 2). *AQP0* and *AQP8* of *P. vlangalii* clustered more closely with those of *X. laevis*, indicating a conserved evolutionary link between reptile and amphibian. In contrast, *AQP1*, *AQP3*, *AQP4*, *AQP5*, *AQP7* and *AQP9* showed higher sequence similarity and co-evolutionary relationships with homologs from *H. sapiens*, *M. musculus* and *X. laevis*. Additionally, *AQP2* and *AQP6* exhibited a closer phylogenetic affinity to *H. sapien* and *M. musculus*. Collectively, apart from *AQP8,* the other AQP genes of *P. vlangalii* show no significant phylogenetic affinity with *C. elegans*.

### 3.4. Predictive Analysis of Secondary and Tertiary Structures

As summarized in Table 3, the secondary structure of the AQP proteins is predominantly composed of α-helix, supporting their role as the structural core of AQPs and their essential function in transmembrane pore formation. In contrast, β-turns account for less than 10% of the residues in most AQPs, suggesting a limited role in local conformational stabilization rather than overall folding. The proportions of random coils and extended strands were generally comparable, which may reflect structural adaptability involved in protein-molecule interactions.

**Figure 3:**
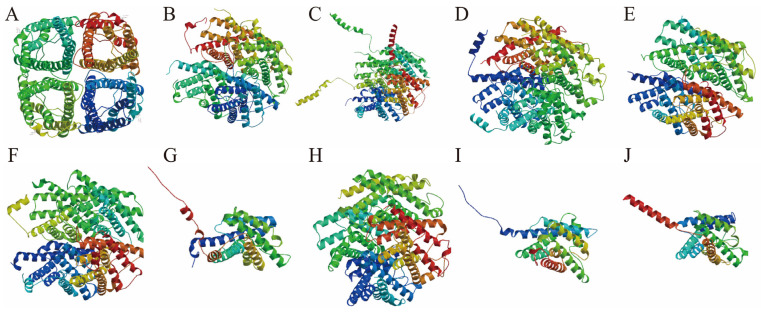
Predicted tertiary structure diagram of AQPs in *P. vlangalii*. The tertiary structures are displayed as follows: *AQP0* (**A**), *AQP1* (**B**), *AQP2* (**C**), *AQP3* (**D**), *AQP4* (**E**), *AQP5* (**F**), *AQP6* (**G**), *AQP7* (**H**), *AQP8* (**I**), and *AQP9* (**J**).

The predicted tertiary structures of the AQP family are illustrated in Figure 3. Each AQP exhibits a distinct spatial architecture, though all members share the presence of multiple transmembrane α-helix. These helices assemble into specific three-dimensional conformations, forming central pores that facilitate the selective passage of water molecules. The highly coiled folding patterns observed in the tertiary models corroborate the secondary structure predictions. While the fractions of random coil and extended strands are relatively low across AQPs, the proportion of α-helix and β-turn angle varies considerably among different members.

To validate the transmembrane topology of the predicted AQP structures, we performed transmembrane helix prediction using TMHMM 2.0. Consistent with the canonical aquaporin fold, nine AQP proteins (*AQP0* to *AQP8*) were predicted to contain six transmembrane helices (TM1-TM6). However, a notable exception was *AQP9*, which was robustly predicted to possess only five transmembrane helices. The underlying amino acid sequence for *P. vlangalii AQP9* was verified, suggesting this may represent a unique structural divergence in this species or lineage.

### 3.5. Expression Profile of AQP Genes in Different Tissues

**Figure 4:**
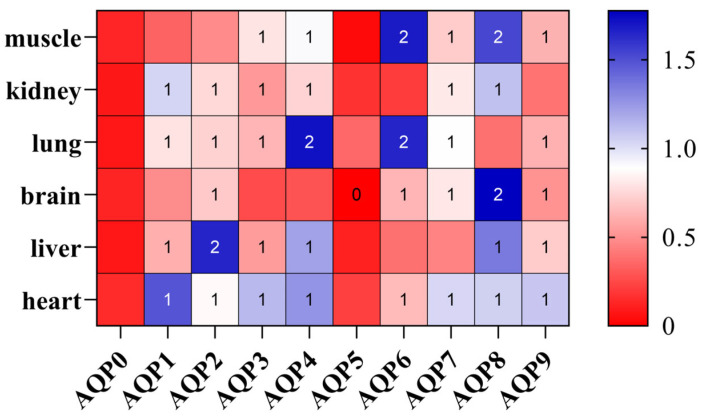
Expression heatmap of the AQP gene family in *P. vlangalii*. Gene-based Z-score normalization. Color intensity represents the relative expression Z-score normalized by row to show the expression pattern of each gene across different tissues, with blue indicating higher expression, while red indicates lower expression in each tissue.

qPCR results (Figure 4) revealed tissue-specific expression patterns of AQP genes in healthy adult individuals of *P. vlangalii*. In the kidney, AQP expression was largely undetectable, with only trace levels observed for *AQP1*, *AQP2*, *AQP3*, *AQP4*, *AQP7*, and *AQP8*. In contrast, muscle and lung tissues exhibited the broadest expression profiles, with most AQP family members detected. Notably, *AQP6* and *AQP8* showed the highest transcript abundance in these tissues. Within muscles, significant expressions were primarily restricted to *AQP6* and *AQP8*. In the brain, *AQP8* was prominently expressed. Brain tissues displayed low but detectable expressions of multiple AQPs, except for *AQP0* and *AQP5*. Liver expression was generally low, with *AQP0*, *AQP5*, *AQP6*, and *AQP7*, primarily representing the classical aquaporin subfamily, which was expressed at reduced levels.

**Figure 5:**
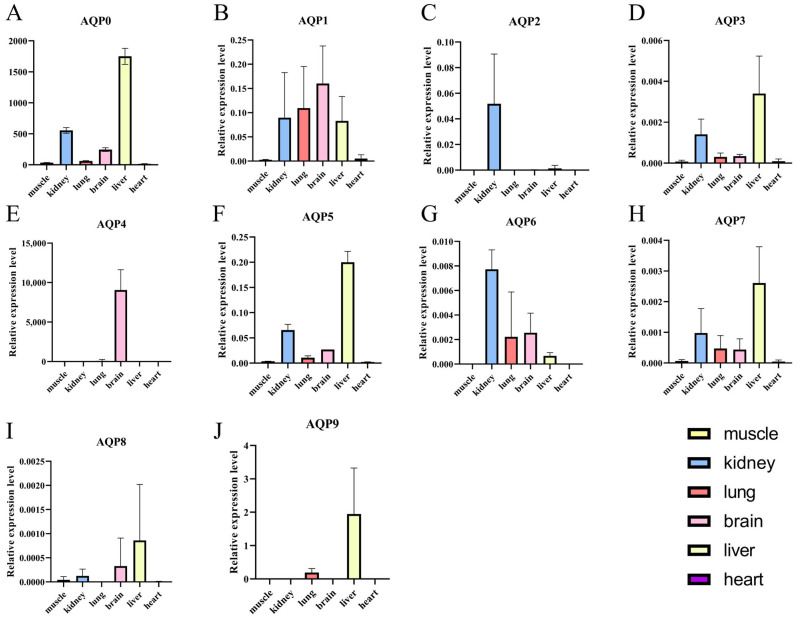
Relative expression levels of AQP genes across various tissues of *P. vlangalii*. (**A**−**J**) represent the expression levels of the *AQP0* to *AQP9* genes in six tissues: heart, kidney, lung, brain, liver, and muscle.Data are presented as means ± SE. Bars represent expression levels of AQP genes in the muscle, kidney, lung, brain, liver and heart of *P. vlangalii*. Tissues are color-coded as follows: kidney (blue), lung (red), brain (pink), liver (yellow), Muscle and heart are shown in grayscale due to their lower expression levels.

As illustrated in Figure 5, AQP proteins also displayed distinct tissue-specific expression. *AQP0* was detected in all tissues except the heart, with prominent expression in the liver. *AQP1* was ubiquitously expressed and highly abundant in the kidney, lung, brain and liver. *AQP2* expression was kidney-specific. *AQP3* was present in all tissues, with the highest levels in the liver and kidney. *AQP4* was exclusively detected in the brain. *AQP5* showed widespread expression, particularly enriched in the kidney and liver. *AQP6* was expressed in the liver, kidney, lung, and brain, with the strongest expression in the kidney. *AQP7* was expressed in all tissues and was most abundant in the liver. *AQP8* and *AQP9* were both predominantly expressed in the liver.

### 3.6. Tissue-Specific Remodeling of AQP Expression in Response to Cold Stress and Recovery

**Figure 6:**
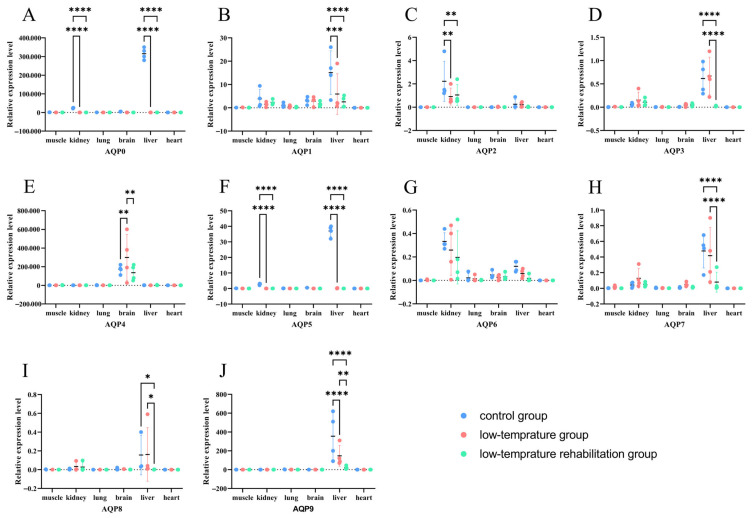
Expression levels of AQP genes across multiple tissues of *P. vlangalii* under different temperature conditions. (n = 4 biologically independent individuals) Data are presented as means ± SE. (**A**–**J**) show the expression profiles for *AQP0* to *AQP9*, respectively. Bars represent expression in muscle, kidney, lung, brain, liver, and heart for the control (blue), low-temperature (pink), and recovery (green) groups. Asterisks indicate statistically significant differences after FDR correction (* *p* < 0.05, ** *p* < 0.01, *** *p* < 0.001, **** *p* < 0.0001).

The expression of multiple AQPs underwent tissue-specific remodeling in coordination with cold stress and the subsequent recovery process (Figure 6). Most notably, *AQP0*, *AQP2*, and *AQP5* were consistently downregulated across multiple tissues during both cold exposure and recovery phases. In contrast, *AQP3*, *AQP4*, and *AQP7* displayed more distinct tissue-specific responses, with significant upregulation observed in certain tissues under cold conditions, followed by downregulation during recovery in others. These results highlight the differential regulatory strategies employed by classical aquaporins versus aquaglyceroporins in response to thermal challenge.

## 4. Discussion

AQPs are a superfamily of major intrinsic proteins that function as channels for water and, in some cases, small neutral solutes like glycerol, playing critical roles in physiological homeostasis across all domains of life [28]. Understanding their genetic and structural features in extremophile species such as *P. vlangalii* offers profound insights into evolutionary adaptation [29,30]. Our analysis is based on foundational research detailing the phylogeny, conserved protein motifs, and genomic structure of the AQP family in *P. vlangalii*. The phylogenetic tree constructed from the amino acid sequences of *P. vlangalii* AQPs resolved into three distinct clades, a topology that is largely consistent with the established classification of aquaporins across the vertebrate subphylum [31]. Extensive comparative genomic studies in fish, amphibians, birds, and mammals consistently recover these major groupings [32,33], indicating that the diversification into water-specific channels and solute-transporting channels occurred early in vertebrate history, long before the divergence of the reptilian lineage. The phylogenetic placement of *AQP0*, *AQP1*, *AQP2*, *AQP4*, and *AQP5* as classical aquaporins and *AQP3*, *AQP7*, and *AQP9* as aquaglyceroporins in *P. vlangalii* is consistent with their functional classifications in species ranging from *Danio rerio* to *H. sapiens*. Furthermore, the phylogenetically distinct *AQP8* clusters within the GlpF superfamily, reflecting its origin as a homolog of bacterial glycerol facilitators [34]. The phylogenetic structure of the AQP family in *P. vlangalii* is not unique but rather a clear illustration of the conserved evolutionary framework seen across vertebrates. This deep conservation underscores the essential and non-redundant physiological roles these protein subfamilies play.

In contrast to the sequence and phylogenetic conservation, the genomic architecture of the *P. vlangalii* AQP genes exhibits considerable diversity. The variation in exon–intron structure among paralogs within a single species is a known phenomenon and has been proposed to be a major driver of regulatory complexity and functional innovation [35]. This variation within a single gene family is not unusual. Comparative genomic studies in other organisms, such as the extensive analysis of the AQP family in *X. laevis*, reveal significant differences in gene number and structure, even between closely related species, driven by events like whole-genome duplication and subsequent gene loss or pseudogenization [36]. The AQP gene family of *P. vlangalii* shares the core characteristics of a vertebrate AQP family, adhering to the conserved principles of phylogeny and protein structure. However, its diverse gene structures point to a distinct evolutionary history at the genomic level, providing a rich area for future research into the regulatory adaptations that enable life in extreme environments.

While AQPs have been well characterized across all major vertebrate classes, Reptilia remain a notable exception [37]. In this study, we compared AQP expression patterns in the kidney, liver, brain, lung, heart, and muscle of *P. vlangalii* with established profiles from other vertebrate lineages, including mammals, birds, amphibians, and fish. Our analysis identified several conserved features, including kidney-specific expression of *AQP2*, prominent expression of *AQP1* and *AQP4* in the brain, and dominant expression of aquaglyceroporins in the liver. Significant differences were also observed, such as a distinct pulmonary AQP profile and unexpected expression of *AQP0* in multiple non-lenticular tissues. The broad expression of multiple AQP isoforms in the kidney of *P. vlangalii* was highly consistent with findings in mammals [38]. The exclusive presence of *AQP2* in the kidney is consistent with a conserved hormonal control mechanism for water reabsorption in amniotes. This interpretation finds support in the expression of *AQP2* in the collecting ducts of birds, which independently evolved the ability to concentrate urine [13]. Together, these findings suggest that the recruitment of *AQP2* for regulated renal water reabsorption represents a key and shared evolutionary step in terrestrial vertebrates. The prominent expression of *AQP1* and *AQP4* in the brain of *P. vlangalii* presents a compelling parallel to their well-established, distinct roles in the mammalian central nervous system, where *AQP1* is involved in cerebrospinal fluid production at the choroid plexus, and *AQP4* facilitates water homeostasis and clearance at the glia–blood interface [39]. The conservation of this expression pattern in a reptilian brain implies that the fundamental molecular machinery for specialized water handling in neural tissues may be an ancient feature of amniote evolution. Based on this conserved molecular architecture, we hypothesize that *AQP4* in *P. vlangalii* may contribute to a form of neuro-fluid clearance. However, it is crucial to note that the existence of a mammalian-like glymphatic system, with its specific astroglial-vascular anatomy, has not been established in reptiles. Therefore, while our data highlight *AQP4* as a key candidate for managing brain water balance, the precise physiological mechanisms in the squamate brain remain an open and fascinating question. Future studies combining detailed neuroanatomy with functional assessments will be essential to determine if a convergent, *AQP4*-dependent clearance pathway operates in reptilian lineages [40]. The co-expression of these two isoforms in the lizard brain is consistent with the view that this functional division is a deeply conserved feature of the amniote brain, likely established before the divergence of synapsid (mammal) and sauropsid (reptile/bird) lineages. In the liver of *P. vlangalii*, expression was dominated by aquaglyceroporins (*AQP3*, *5*, *7*, *8*, *9*), which facilitate the transport of glycerol and other small solutes in addition to water. This pattern is strongly conserved in mammals, where aquaglyceroporins, particularly *AQP9*, are highly expressed in hepatocytes and are crucial for glycerol uptake from the blood for gluconeogenesis [41]. The high expression of this AQP subclass in the lizard liver is consistent with a conserved role in metabolic physiology across vertebrates.

An unexpected finding in *P. vlangalii* was the prominent transcript expression of *AQP0* in the liver. In virtually all other vertebrates studied, *AQP0* has been characterized almost exclusively as a structural protein and water channel specific to ocular lens fiber cells [42]. Its abundant transcript expressions in a major metabolic organ such as the liver represents a notable difference from this canonical view and could indicate a novel, non-lenticular role for this protein in squamate reptiles, a possibility that merits further investigation. Additionally, the gene structure analysis revealed a notably compact architecture for *AQP5* compared to other AQPs in *P. vlangalii*. In general, such reduced intronic complexity has been proposed to be associated with more constrained transcriptional regulation and potentially faster transcript processing. While the functional implications of this structural feature in reptiles are not yet defined, its conservation could reflect a significant, lineage-specific evolutionary constraint. Given the fundamental anatomical differences between the mammalian alveolar lung and the reptilian faveolar lung, the role of *AQP5* in *P. vlangalii* may be distinct from its established function in alveolar fluid secretion. Future investigations into the subcellular localization and physiological regulation of *AQP5* in the reptilian respiratory system are warranted to elucidate its unique role in this context [43].

AQPs are integral membrane proteins that form channels for the transport of water and, in some cases, other small solutes across biological membranes [44,45]. Their regulation is essential for maintaining cellular homeostasis, particularly under abiotic stress conditions such as cold [46]. In this study, we observed a clear divergence in the expression response between two major AQP subfamilies: a consistent downregulation of classical AQPs (e.g., *AQP0*, *AQP2*, *AQP5*) and a variable, tissue-specific upregulation of aquaglyceroporins (e.g., *AQP3*, *AQP7*) as well as other AQPs such as *AQP4*.

The widespread downregulation of classical AQPs across multiple tissues during both cold exposure and recovery likely represents a highly conserved protective physiological response. This strategy could confer advantages such as preventing cellular dehydration and mitigating cold-related damage. Downregulating water channels could reduce water efflux, thereby conserving intracellular water under osmotic stress [47]. Moreover, under low-temperature conditions, limiting AQP-facilitated water flux might slow the growth of potentially damaging extracellular ice crystals [48]. By reducing the number of functional water channels, cells might slow such processes, which could be advantageous. The mechanisms underlying this downregulation are complex and involve multiple regulatory layers. Cold stress is known to trigger signaling cascades that recruit repressive transcription factors in other systems [49]. Beyond transcriptional control, AQP activity can be rapidly modulated by post-translational modifications such as phosphorylation, which can gate the channel pore and reduce water permeability independently of transcript levels [50]. The persistence of downregulation into the recovery phase is consistent with a model in which re-establishment of water transport homeostasis is a gradual process, potentially protecting against osmotic shock during rewarming.

In contrast, the upregulation of aquaglyceroporins such as *AQP3* and *AQP7* in specific tissues reflects an active, adaptive metabolic strategy. This response may be driven by tissue-specific demands for glycerol transport, primarily for cryoprotection and energy metabolism. Many cold-tolerant organisms accumulate high glycerol concentrations in tissues and body fluids, which can act as a cryoprotectant to mitigate low-temperature damage [51]. Studies in freeze-tolerant frogs have suggested that cold-induced aquaglyceroporin upregulation facilitates necessary glycerol fluxes [21]. Cold exposure can also increase metabolic rate and energy demand, particularly in specialized tissues such as brown adipose tissue (BAT). Glycerol released from triglyceride breakdown in adipose tissue serves as a key substrate for this process [52]. *AQP7*, known as a channel for glycerol efflux from adipocytes in mammals [53], exhibits cold-inducible expression in BAT, supporting its role in mobilizing fuel for thermogenesis. Similarly, *AQP3* and *AQP9* are implicated in glycerol uptake and metabolism in other tissues such as the liver [52,54]. Thus, the tissue-specificity of aquaglyceroporin upregulation is closely linked to the metabolic role of the tissue under cold stress.

Notably, *AQP4*, a classical aquaporin, exhibited a distinct response, indicating that tissue-specific function can override general subfamily trends. We propose that the regulation of *AQP4* might be adopted to help maintain brain water homeostasis, adopting a strategy distinct from the broad downregulation seen for other classical AQPs. During recovery, aquaglyceroporins were downregulated, consistent with a return to baseline homeostasis. This likely corresponds to a decreased demand for glycerol transport for cryoprotection and thermogenesis declines. Attenuation of cold-activated signaling pathways, including specific transcription factors and hormonal cascades, likely leads to reduced *AQP3* and *AQP7* expression back to pre-stress levels, supporting the dynamic nature of this response. Although the signaling pathways involved in recovery remain poorly understood, they may include those that restore metabolic and osmotic balance, such as the MAPK and NF-κB pathways, analogous to mechanisms observed in ischemia–reperfusion contexts [55].

In summary, the tissue-specific remodeling of AQP expression during cold stress and recovery is consistent with a highly coordinated, function-driven strategy that leverages the distinct physiological roles of AQP subfamilies. Classical AQPs (e.g., *AQP0*, *AQP2*, *AQP5*) are consistently downregulated as a fundamental protective mechanism to conserve cellular water and mitigate cold damage, a strategy that appears to be a broadly conserved response to abiotic stress. In contrast, aquaglyceroporins (e.g., *AQP3*, *AQP7*) are dynamically upregulated in a tissue-specific manner to manage metabolic demands, facilitating glycerol transport for cryoprotection and as an energy substrate for thermogenesis. This differential regulation is hypothesized to be initiated by cold-sensing mechanisms, likely involving TRP channels such as TRPM8, which can activate divergent downstream signaling cascades [56]. These cascades are thought to engage distinct sets of transcription factors, hormonal signals, and post-translational modifications tailored to either suppress water transport or enhance solute transport according to tissue-specific metabolic requirements [57]. Together, these divergent regulations illustrate a coordinated transcriptional response in *P. vlangalii*, suggesting an integration of protective and metabolic adjustments through differential control of the aquaporin family.

## 5. Limitations of the Study

This study has a limitation in its sample size (n = 4 per group), which is small. This is due to the challenges associated with collecting this protected plateau-endemic species under strict permits, coupled with the extreme logistical difficulties in obtaining specimens from remote high-altitude locations. We acknowledge this limitation, and future studies with larger sample sizes would be valuable to confirm and extend our findings. However, through rigorous experimental controls, the application of robust statistical models (linear mixed-effects models and FDR), and false discovery rate correction, we are confident that the major expression trends reported are reliable and biologically meaningful. Future studies with larger sample sizes would be valuable to further corroborate these findings.

While we confirmed primer specificity through melt curve analysis and amplicon size validation, our qPCR analysis lacked formal standard curve data for amplification efficiency and no-RT controls in the final experimental runs, representing a deviation from the highest MIQE guidelines. Finally, this study focused on transcriptional regulation; however, changes at the protein level and channel activity are likely more direct. Thus, our findings offer crucial preliminary insights into the molecular mechanisms of cold adaptation, and subsequent functional validation experiments will be essential.

## 6. Conclusions

In this study, we identified a total of 10 AQP family genes in *P. vlangalii* and comprehensively characterized their phylogenetic relationships and structural features. Tissue-specific expression profiling revealed that these AQPs are distributed across multiple organs and are involved in distinct regulatory processes, which may underpin the physiological adaptability of *P. vlangalii* to extreme plateau conditions. Furthermore, an investigation into the species’ response to cold stress and recovery uncovered a tissue-specific regulatory mechanism that enhances its environmental resilience. Within the limits of this study, these findings not only represent a significant advance in the understanding of *P. vlangalii* biology but also offer broader insights into the adaptive strategies of plateau-dwelling vertebrates.

## Figures and Tables

**Table 1:** AQP gene family specific primers.

Gene	Primer Sequences	Tm/°C	Size/bp
*PvlAQP0*	F:CCCGCAACTTCACCAACCT R:AGCTGCTCACCTTTCAAGATAGAG	56.30	142
*PvlAQP1*	F:GCCACAACAGACAGGAGGAGA R:TACACAGCGGAGCCGAAAG	60.10	143
*PvlAQP2*	F:TAGGTTCCCAAGTCTCCTTCCTT R:AGAACTTCCTCGGATGTGGTGT	56.30	119
*PvlAQP3*	F:ATGCCTGGGAACCCTCGTA R:AATGCCAAGCGTCACAGCA	55.40	130
*PvlAQP4*	F:TTCTTGCCGGAACCCTTTAT R:GGCTCCTGGTATCTTCTACTTCTATG	43.90	132
*PvlAQP5*	F:TCTGCTCAATGGGTCTTCTGG R:GACTCGTAGGTGCCTTTCACG	53.00	134
*PvlAQP6*	F:CTCGTATCTCCCTGGTGAAAGC R:GAGTTCTGAATCATGTTGATGCCTA	58.80	136
*PvlAQP7*	F:GACTTGCACAATCGGTCTCG R:CGTCTTGCTTCCTACATTCCTC	52.40	70
*PvlAQP8*	F:CTCTTGGTGCCTGGCTCATT R:TGCGACCGTCACAAACTGC	63.50	137
*PvlAQP9*	F:AGCCCTGGGTGCGGTC R:GGTCAAGTTCGTGCTGGTTTC	57.60	92
*ACTB*	F:CACGGCATTATCACTAACTGGG R:GTAAGGTGGGATGTTCCTCTGG	43.30	97

**Table 2:** Characteristics of the AQP gene family in the genome of *P. vlangalii*.

ID	Gene Name	Number of Amino Acid	Molecular Weight (Da)	pI	GRAVY	Instability Index	Aliphatic Index
GWHPAAFC012688	*PvlAQP0*	263	28227.04	6.97	0.645	33.50	112.74
GWHPAAFC017642	*PvlAQP1*	269	28459.04	6.06	0.556	36.04	109.85
GWHPAAFC016973	*PvlAQP2*	273	29846.73	6.83	0.428	47.92	106.15
GWHPAAFC015342	*PvlAQP3*	292	31452.68	6.70	0.498	24.51	104.25
GWHPAAFC011293	*PvlAQP4*	312	33801.56	6.81	0.446	28.63	108.75
GWHPAAFC016972	*PvlAQP5*	261	28240.04	6.95	0.548	39.51	98.62
GWHPAAFC012707	*PvlAQP6*	264	28168.94	7.77	0.688	39.03	120.53
GWHPAAFC015346	*PvlAQP7*	271	28776.54	6.40	0.552	37.05	107.31
GWHPAAFC014848	*PvlAQP8*	243	26088.66	5.61	0.572	35.38	111.98
GWHPAAFC012538	*PvlAQP9*	263	27874.82	6.22	0.543	37.68	109.13

**Table 3:** Secondary structure prediction of AQP proteins.

Gene ID	Protein Name	Alpha Helix (%)	Beta Turn (%)	Random Coil (%)	Extended Strand (%)
GWHPAAFC012688	*PvlAQP0*	39.92	2.66	36.88	20.53
GWHPAAFC017642	*PvlAQP1*	38.29	1.86	40.52	19.33
GWHPAAFC016973	*PvlAQP2*	41.76	2.93	34.43	20.88
GWHPAAFC015342	*PvlAQP3*	38.01	2.74	36.99	22.26
GWHPAAFC011293	*PvlAQP4*	36.54	2.24	41.67	19.55
GWHPAAFC016972	*PvlAQP5*	41.76	3.07	36.40	18.77
GWHPAAFC012707	*PvlAQP6*	43.94	1.52	37.12	17.42
GWHPAAFC015346	*PvlAQP7*	38.01	2.74	36.99	22.26
GWHPAAFC014848	*PvlAQP8*	46.50	3.70	31.69	18.11
GWHPAAFC012538	*PvlAQP9*	32.32	3.80	38.02	25.86

## Data Availability

The datasets supporting the conclusions of this article are available in the Supplementary Materials from the Figshare repository (DOI: 10.6084/m9.figshare.30636053.v1) and the China National Center for Bioinformation (CNCB).

## Supplementary Materials

The following supporting information can be downloaded at https://www.mdpi.com/article/10.3390/biology14121755/s1, Table S1. Tertiary structure characteristics of proteins in *P. vlangalii*. Table S2. BLAST alignment analysis of five species. Table S3. Original data for qPCR. Table S4. Original data for ANOVA. Figure S1. Melting curve analysis for reference and AQP genes.
