# Supplementary material for: Genome-Wide Identification and Expression Analysis of the Aquaporin Gene Family in the Qinghai Toad-Headed Agama (Phrynocephalus vlangalii) and Responses to Acute Cold Stress
Source: Biology (Basel). 2025 Dec 7;14(12):1755. doi: 10.3390/biology14121755 (PMC12731239; doi:10.3390/biology14121755)
Supplement: Supplementary file 1 [file biology-14-01755-s001.zip › Supplement.pdf]

Table S1. Tertiary structure characteristics of proteins in P. vlangalii

Gene	Template PDB	Seq Identity (%)	Coverage	GMQE	QMEAN
PvlAQP0	1ymg.1.D	73.38	1.00	0.82	0.81 ± 0.05
PvlAQP1	7uze.1.A	84.27	0.99	0.77	0.76 ± 0.05
PvlAQP2	4oj2.1.A	75.94	0.97	0.79	0.75 ± 0.05
PvlAQP3	8y8n.1.D	80.76	1.00	0.85	0.83 ± 0.05
PvlAQP4	8v8s.1.A	83.65	1.00	0.70	0.86 ± 0.05
PvlAQP5	5c5x.1.A	74.90	0.93	0.79	0.79 ± 0.05
PvlAQP6	A0A670YDS8.1.A	74.62	1.00	0.92	-
PvlAQP7	8y8v.1.A	57.44	0.89	0.74	0.78 ± 0.05
PvlAQP8	A0A4D9ETD2.1.A	75.85	0.97	0.93	-
PvlAQP9	A0A6J0SVS7.1.A	86.26	1.00	0.93	-

Table S2. BLAST alignment analysis of five species

Gene	species	accession numbers
PvlAQP0	Homo sapiens	NP_036196.1
PvlAQP0	Gallus gallus	NP_989597.1
PvlAQP0	Xenopus laevis	NP_001088304.1
PvlAQP0	Mus musculus	NP_032626.2
PvlAQP0	Protopterus annectens	BAH98061.1
PvlAQP1	Homo sapiens	BAG70089.1
PvlAQP1	Mus musculus	AAH07125.1
PvlAQP1	Bos taurus	NP_777127.1
PvlAQP1	Gallus gallus	NP_001034542.1
PvlAQP1	Ovibos moschatus	KAL1288347.1
PvlAQP2	Homo sapiens	AAB31999.1
PvlAQP2	Bos taurus	AAI51513.1
PvlAQP2	Rattus norvegicus	NP_037041.3
PvlAQP2	Xenopus laevis	NP_001079331.1
PvlAQP2	Gekko kuhli	KAL8163911.1
PvlAQP3	Bos grunniens	URM60750.1
PvlAQP3	Homo sapiens	CAG46788.1
PvlAQP3	Marmota monax	KAI6063786.1
PvlAQP3	Gulo gulo luscus	KAI5767167.1
PvlAQP3	Phodopus roborovskii	CAH6790258.1
PvlAQP4	Homo sapiens	AAB26958.1
PvlAQP4	Mus musculus	CAJ18569.1
PvlAQP4	Penaeus japonicus	WHF59464.1
PvlAQP4	Danio rerio	NP_001345242.1
PvlAQP4	Pogona vitticeps	XP_072854984.1
PvlAQP5	Homo sapiens	CAG46786.1
PvlAQP5	Mus musculus	NP_033831.1
PvlAQP5	Xenopus tropicalis	NP_001297041.1

PvlAQP5	Bufotes viridis	CAN1979325.1
PvlAQP5	Gallus gallus	XP_040549747.1
PvlAQP6	Mus musculus	AAI15587.1
PvlAQP6	Homo sapiens	NP_001643.2
PvlAQP6	Bos taurus	XP_002687325.1
PvlAQP6	Pelodiscus sinensis	XP_075758186.1
PvlAQP6	Lepidochelys kempii	XP_073174757.1
PvlAQP7	Homo sapiens	AAI19674.1
PvlAQP7	Phodopus roborovskii	CAH6790257.1
PvlAQP7	Gobiocypris rarus	ATX74160.1
PvlAQP7	Bos grunniens	URP23533.1
PvlAQP7	Pygoscelis papua	KAK0686832.1
PvlAQP8	Bufotes viridis	CAN2007459.1
PvlAQP8	Bos taurus	AAI16017.1
PvlAQP8	Homo sapiens	AAH40630.1
PvlAQP8	Xenopus tropicalis	AAI58264.1
PvlAQP8	Mus musculus	AAD55972.1
PvlAQP9	Mus musculus	CAJ18382.1
PvlAQP9	Bufotes viridis	CAN1970836.1
PvlAQP9	Homo sapiens	AAH26258.1
PvlAQP9	Paroedura picta	XP_077174121.1
PvlAQP9	Bos mutus	AJE25659.1

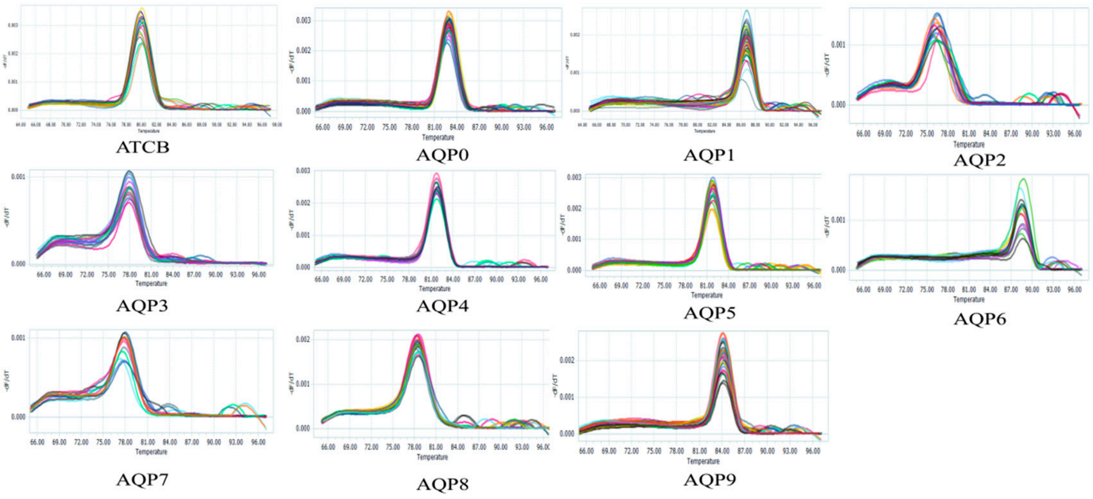

Figure S1. Melting curve analysis for reference and AQP genes
